# Trends in antibiotic resistance of *Streptococcus pneumoniae* and *Haemophilus influenzae* isolated from nasopharyngeal flora in children with acute otitis media in France before and after 13 valent pneumococcal conjugate vaccine introduction

**DOI:** 10.1186/s12879-015-0978-9

**Published:** 2015-06-21

**Authors:** François Angoulvant, Robert Cohen, Catherine Doit, Annie Elbez, Andreas Werner, Stéphane Béchet, Stéphane Bonacorsi, Emmanuelle Varon, Corinne Levy

**Affiliations:** Service d’Accueil des Urgences Pédiatriques, AP-HP, Hôpital Necker-Enfants-Malades, Université Paris Descartes Sorbonne Paris Cité, ECEVE - INSERM UMR1123, Paris, France; ACTIV, Association Clinique et Thérapeutique Infantile du Val de Marne, Saint-Maur des Fossés, France; Clinical Research Center (CRC), Centre Hospitalier Intercommunal de Créteil, Créteil, France; Unité Court Séjour, Petits Nourrissons, Service de Néonatologie, Centre Hospitalier Intercommunal de Créteil, Créteil, France; AFPA, Association Française de Pédiatrie Ambulatoire, Chambéry, France; Université Paris Diderot Sorbonne Paris Cité, Paris, France; Service de Microbiologie, AP-HP, Hôpital Robert-Debré, 75019 Paris, France; National Reference Center for Pneumococci, Laboratoire de Microbiologie, AP-HP, Hopital Européen Georges-Pompidou, Paris, France

**Keywords:** *Streptococcus pneumoniae*, Conjugate vaccine, PCV, Otitis media, Antibiotic, Guideline, Blnar, Betalactamase, *Haemophilus influenzae*

## Abstract

**Background:**

After the implementation of pneumococcal conjugate vaccines (PCVs), the marked shift in *Streptococcus pneumoniae* (Pnc) serotype distribution led to a modification in pneumococcal antibiotic susceptibility. In 2011, the pattern of antibiotic prescription in France for acute otitis media in infants was greatly modified, with decreased use of third-generation cephalosporins and amoxicillin–clavulanate replaced by amoxicillin alone. To assess antibiotic strategies, here we measured the antibiotic susceptibility of Pnc and *Haemophilus influenzae* (Hi) isolated from nasopharyngeal flora in infants with acute otitis media in the 13-valent PCV (PCV13) era in France.

**Methods:**

From November 2006 to June 2013, 77 pediatricians obtained nasopharyngeal swabs from infants (6 to 24 months old) with acute otitis media. The swabs were sent for analysis to the national reference centre for pneumococci in France. Demographics, medical history, and physical examination findings were recorded.

**Results:**

We examined data for 7200 children, 3498 in the pre-PCV13 period (2006–2009) and 3702 in the post-PCV13 period (2010–2013). The Pnc carriage rate decreased from 57.9 % to 54.2 % between the 2 periods, and the proportion of pneumococcal strains with reduced susceptibility to penicillin or resistant to penicillin decreased from 47.1 % to 39 % (P < 0.0001). The Hi carriage rate increased from 48.2 % to 52.4 %, with the proportion of ß-lactamase–producing strains decreasing from 17.1 % to 11.9 % and the proportion of ß-lactamase–nonproducing, ampicillin-resistant strains remaining stable, from 7.7 % to 8.2 %. We did not identify any risk factor associated with carriage of ß-lactamase–producing Hi strains (such as daycare center attendance, otitis-prone condition or recent antibiotic use).

**Conclusion:**

In France, the nasopharyngeal carriage rate of reduced-susceptibility pneumococcal strains and ß-lactamase–producing Hi strains decreased in children with acute otitis media after 2010, the year the PCV13 was introduced. Accordingly, amoxicillin as the first-line drug for acute otitis media requiring antibiotics remains a valid choice.

## Background

Acute otitis media (AOM) is the most frequent bacterial infection in childhood and one of the major indications for antibiotics in many countries [1]. *Streptococcus pneumoniae* (Pnc) and *Haemophilus influenzae* (Hi) are the leading bacterial species implicated in AOM, followed by *Moraxella catarrhalis* (Mc) and, to a lesser extent, group A β-hemolytic streptococci [2]. For AOM, Pnc and Hi are the main targets of antibiotics prescription [3, 4]. Detection of bacterial species in the middle ear fluid (MEF) by culture is the “gold standard” for etiologic diagnosis of AOM [5].

Few medical centers in Europe or the United States continue to routinely perform tympanocentesis [6, 7]. The decrease in centers performing tympanocentesis probably reflects no new antibiotics introduced for AOM [5]. Tympanocentesis or myringotomy (both considered painful) are performed mainly for special circumstances: antibiotics non-response, recurrent cases and chronic OM. Therefore, the profile of antibiotics resistance among otopathogens isolated from MEF could be biased and we have no current MEF microbiology data on which to base treatment decisions for the most frequent situations: first-line AOM seen by pediatricians or general practitioners. Because the microbiology of AOM is linked to nasopharyngeal (NP) flora, many studies have assessed the possibility of using NP culture, a painless procedure, to predict the bacterial etiology of AOM [5, 8]. These studies have shown that if Pnc or Hi is not found in the nasopharynx, the isolates will not likely be found in the MEF in case of AOM. However, because the causative bacteria of AOM are often isolated from NP cultures in addition to other organisms, the positive predictive value of this type of sample for the causative agent is poor [9].

For these reasons, NP culture has not been considered a useful predictor of AOM etiology in clinical practice. However, if NP cultures are not useful for treatment for specific patients with AOM, they may be useful on a population basis for formulating recommendations in one country or in different regions [5].

In France, 7 valent pneumococcal conjugate vaccine (PCV7) was introduced for high risk children < 2 years old in 2002, then for all children < 2 years old in 2006. PCV7 coverage reached 86 % in 2008. In June 2010, the French authorities recommended routine vaccination with PCV13 of infants at 2, 4 and 12 months old to replace PCV7. During a one-year transition period, switching from PCV7 to PCV13 was recommended at any point in the schedule to complete the immunization series. The PCV vaccination coverage for children younger than 2 years after changing from PCV7 to PCV13 is greater than 92 % [10].

Since the implementation of PCV7 and PCV13, two major and related changes have occurred: the Pnc serotype distribution has shifted, which has led to a modification in pneumococcal antibiotic susceptibility. The changes in antibiotic susceptibility could also result from other factors such as variations in antibiotic consumption (volume and type of compound used) or rate of children cared for outside the home [3, 4, 11]

Since 2000, to follow the changes induced by PCV implementation, our research group has analyzed several hundred NP samples from children with AOM all over France each year [12]. The data obtained during the first years of the study have served as the basis for the guidelines published in 2011 for France [3, 13], which recommended amoxicillin, 80–90 mg/kg/d in 2 or 3 daily intakes, as first-line therapy for AOM in young children. Indeed, the guidelines stated that amoxicillin was the most active agent for pneumococci with decreased susceptibility to penicillin and was active for more than 85 % of nontypable Hi according to microbiologic data at the time [3]. Since 2011, 2 major changes have occurred: the implementation of 13-valent PCV (PCV13) and the shift in antibiotic prescription for AOM, with decreased use of third-generation cephalosporins and amoxicillin–clavulanate replaced by amoxicillin [14, 15].

A rapid change in resistance of Pnc and Hi to the commonly used antimicrobials prompts a reevaluation of the treatment strategies [8]. To assess this question, we measured NP carriage and antibiotic susceptibility of these 2 otopathogens in young children with AOM in the PCV13 era in France.

## Methods

From November 2006 to June 2013, 77 French pediatricians throughout France took part in a cross-sectional study. An NP swab was obtained from children aged 6 to 24 months with a diagnosis of purulent AOM [16]. We excluded children who had received antibiotics within 7 days before enrolment, had a severe underlying health disorder, or had been included in the study during the previous 12 months. Demographic data, medical history and physical findings were recorded. To monitor the impact of the introduction of PCV13 on this ecological niche, 2 periods were defined: pre-PCV13 (2006–2009) and post-PCV13 (2010–2013).

The study was approved by the Saint Germain en Laye Ethics Committee (CPP île de France XI, 20 rue Armagis, 78105 Saint Germain en Laye Cedex, France), and written informed consent was obtained from parents or guardians.

### Microbiology investigations

NP specimens were obtained with use of cotton-tipped wire swabs. The swabs were inserted into the anterior nares, gently rubbed on the NP wall and immediately placed in transport medium (Copan Venturi Transystem, Brescia, Italy). The samples were transferred within 48 h to Robert Debré Hospital and to the national reference center for pneumococci in France.

Pnc culture, identification, serotyping and antibiotic susceptibility testing were performed as previously described [17]. Susceptibility of Pnc isolates to penicillin G and erythromycin was determined from minimal inhibitory concentrations (MICs) by the agar-dilution method. Isolates were classified as penicillin-susceptible (MIC ≤ 0.06 μg/ml), penicillin–non-susceptible (MIC ≥ 0.12 μg/ml), penicillin–intermediate-resistant (MIC 0.12–2.0 μg/ml), or penicillin-resistant (MIC > 2 μg/ml) according to the European Committee on Antimicrobial Susceptibility Testing (http://www.eucast.org/clinical_breakpoints/).

Isolates of Hi were identified by colony morphology assay and conventional methods of determination. Hi isolates underwent capsular serotyping by the slide agglutination method with specific antisera (Phadebact; Boule Diagnostics, Huddinge, Sweden). The production of ß-lactamase was assessed by a chromogenic cephalosporin test (Nitrocefin; Cefinase; Biomerieux, Marcy l’Etoile, France). *H. influenzae* strains were further classified as ampicillin susceptible (MIC ≤ 1 mg/L), or resistant (MIC > 1 mg/L). ß-lactamase negative ampicillin resistance (BLNAR) was determined according to the CLSI break points [18]. If the 2 μg ampicillin diffusion test (Becton Dickinson) gave a zone of inhibition <20 mm and if the cefalotin disk diffusion test gave a zone of inhibition <17 mm, strains were considered BLNAR [19].

### Statistical analysis

Data were double-entered by using 4D software 6.4 and analyzed by using Stata SE 13.1 (Stata Corp., College Station, TX, USA) for univariate analysis and multivariate logistic regression (odds ratios [ORs] and 95 % confidence intervals [95 % CI]). Chi-square test was used to compare NP carriage of pneumococci and Hi before and after PCV13 implementation. Factors related to NP carriage were identified on univariate analysis (p < 0.20, chi-square test). Multivariate logistic regression analysis was performed to identify the main factors associated with carriage of Pnc, Pnc strains with reduced susceptibility to penicillin (RSP), Hi carriage, and ß-lactamase–producing Hi strains. For these models, factors had to be determined by the physician during the visits. When different factors were highly correlated, such as recent antibiotic use, history of AOM and otitis-prone children, we retained the most relevant factor. The following variables were included in multivariate logistic regression models: daycare attendance, siblings, recent antibiotic treatment (within 3 months before enrolment), otalgia + fever ≥38.5 °C, conjunctivitis, and bilateral AOM. All models were adjusted for age. Age was dichotomized as <1 and ≥ 1 year. The cut-off of 1 year was chosen because the vaccination schedules differ before and after this age (reflecting the immunity maturation); in many studies, the NP carriage is higher after 1 year; and in one of our studies, young age (<1 year) predicted penicillin non-susceptible pneumococci carriage [20].

## Results

During the 7 years, we assessed samples for 7200 patients (3498 in the pre-PCV13 period and 3702 in the post-PCV13 period). Table 1 shows demographic characteristics and NP carriage of the children enrolled. Median age was 13 months (Q1–Q3 9–17 months) and more than 99 % were PCV-vaccinated, including 3399 (47.2 %) with PCV13. Among the children, 44.8 % attended a daycare center and 45.1 % had received antibiotics within 3 months before inclusion.

**Table 1:** Characteristics of children with acute otitis media before/after 13-valent pneumococcal conjugate vaccine implementation in France

Child characteristics	Before PCV13	After PCV13	p-value
	*n* = 3,498 (%)	*n* = 3,702 (%)	
Male sex	1,848 (52.8)	1,982 (53.5)	0.5
Age (months), mean ± SD	13.5 ± 5.0	13.7 ± 5.0	0.05
Median (Q1-Q3)	12.7 (9.4-17.2)	13.0 (9.5-17.5)	
Type of care			<0.0001
Daycare center	1437 (41.1)	1784 (48.2)	
Childminder	1099 (31.4)	1148 (31.0)	
Home	959 (27.4)	770 (20.8)	
Antibiotics 3 months before enrolment	1652 (47.3)	1591 (43.0)	<0.0001
Only 1 antibiotic	1035 (62.7)	1051 (66.1)	0.04
At least 2 antibiotics	617 (37.4)	539 (33.9)	
Cephalosporin	809 (49.1)	393 (24.8)	
Amoxicillin-clavulanate	670 (40.7)	561 (35.4)	<0.0001
Amoxicillin	116 (7.0)	598 (37.7)	
Others antibiotic	52 (3.2)	34 (2.1)	
Last antibiotic in			
the previous month	607 (36.7)	601 (37.8)	
1 to 2 months	681 (41.2)	666 (41.9)	0.50
2 to 3 months	364 (22.0)	324 (20.4)	
Sibling	1975 (56.5)	2071 (55.9)	0.65
Conjunctivitis	886 (25.3)	1062 (28.7)	0.001
Otalgia	2595 (74.3)	2686 (72.6)	0.1
Fever (≥38.5 °C)	2061 (59.4)	2052 (55.9)	0.003
Otalgia + fever ≥38.5 °C	1564 (44.7)	1543 (41.7)	0.009
Bilateral^a^	492/900 (54.7)	1858 (50.2)	0.02
History of AOM^b^	1005/1793 (56.1)	2047 (55.3)	0.6
Otitis-prone children^b^	349/1793 (19.5)	651 (17.6)	0.09
Pnc carriage	2026 (57.9)	2007 (54.2)	0.002
Hi carriage	1686 (48.2)	1938 (52.4)	<0.0001

^a^data not available in 2006/2008, ^b^data not available in 2006/2007

AOM: Acute Otitis Media; PCV13: 13-valent pneumococcal conjugate vaccine; Q1-Q3, quartiles 1 to 3; Pnc: *pneumococcus*; Hi*: H. influenzae*

**Fig. 1:**
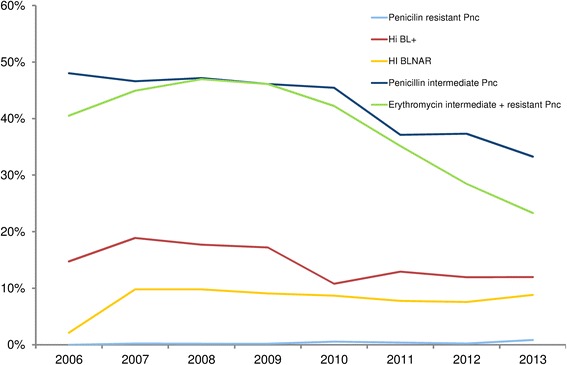
Antibiotic resistance of *Streptococcus pneumoniae* and *Haemophilus influenzae* isolated from nasopharyngeal flora in 7200 infants with acute otitis media between 2006 and 2013. Pnc: *pneumococcus*; Hi*: H. influenzae*; BL+: ß-lactamase–producing strains; BLNAR: ß-lactamase–nonproducing ampicillin-resistant strains

The Pnc carriage rate decreased from 57.9 % to 54.2 % between the pre- and post-PCV13 period, and the proportion of Pnc strains with reduced susceptibility to penicillin or resistant to penicillin decreased from 47.1 % to 39 % (P < 0.0001) (Table 2 and Fig. 1). In the pre-PCV13 period, pneumococcal penicillin–non-susceptible strains were represented by serotypes 19A, 15A, 35B and 19 F. Among the Pnc carriers, between the two periods, the proportion of PCV7 serotype and 6 additional PCV13 serotypes decreased from 9.5 % to 3.2 % (P < 0.001) and from 32.0 % to 11.1 % (P < 0.001), respectively. Serotype 19A, the most frequently serotype identified during the whole study, decreased from 22.6 % to 8.4 % between the 2 periods. By contrast, non-PCV13 serotypes increased from 58.5 % to 85.7 % (P < 0.001). The most frequently carried non-PCV13 serotypes in post PCV13 period were serotype 15B/C (12.2 %), 15A (9.3 %), 11A (9.1 %), 35B (7.5 %), 23A (5.9 %), and 6C (5.0 %), 33 other serotypes accounted for 34.9 %. In this period, pneumococcal penicillin–non-susceptible strains were predominantly represented by serotypes 11A, 15A, 15B/C, 19A and 35B. Non-typeable Pnc remained stable (2.1 % to 1.8 %) between the 2 periods.

**Table 2:** Nasopharyngeal carriage and resistance of Pnc and Hi in children with AOM before/after PCV13 implementation

Carriage	Before PCV13	After PCV13	p-value
	*n* = 3,498 (%)	*n* = 3,702 (%)	
**Pnc**	2026 (57.9)	2007 (54.2)	
Penicillin susceptible	1069 (52.9)	1223 (61.0)	<0.001
Penicillin intermediate-resistant	950 (47.0)	772 (38.5)	
Penicillin resistant	3 (0.1)	10 (0.5)	
Erythromycin susceptible	1119 (55.3)	1351 (67.4)	<0.001
Erythromycin intermediate	109 (5.4)	42 (2.1)	
Erythromycin resistant	794 (39.3)	613 (30.6)	
PCV7 vaccine types	193(9.5)	64 (3.2)	<0.001
Additional PCV13 vaccine types	648 (32.0)	223 (11.1)	
Non-vaccine types	1185 (58.5)	1720 (85.7)	
**Hi**	1686 (48.2)	1938 (52.4)	
ß-lactamase–producing strains	289 (17.1)	230 (11.9)	<0.001
BLNAR+	130 (7.7)	159 (8.2)	0.74
ß-lactamase + BLNAR-	264 (15.7)	209 (10.8)	
ß-lactamase + BLNAR+	25 (1.4)	21 (1.1)	
ß-lactamase- BLNAR+	105 (6.2)	138 (7.1)	
ß-lactamase- BLNAR-	1292 (76.6)	1570 (81.0)	
No carriage	693 (19.8)	773 (20.9)	
Hi or Pnc carriage	1898 (54.3)	1913 (51.7)	0.09
Hi and Pnc carriage	907 (25.9)	1016 (27.4)	

Pnc: *pneumococcus*; Hi*: H. influenzae*; BLNAR: ß-lactamase–nonproducing ampicillin-resistant

The Hi carriage rate increased from 48.2 % to 52.4 % between the pre- and post-PCV13 period. During the whole study, 18/3624 (0.5 %) Hi isolates were serotype b. The proportion of ß-lactamase–producing Hi strains decreased from 17.1 % (*n* = 289) to 11.9 % (*n* = 230) and that of ß-lactamase–nonproducing, ampicillin-resistant (BLNAR) strains remained stable, < 10 %, with 7.7 % and 8.2 % in the pre- and post-PCV13 periods (Table 2).

On multivariate logistic regression analysis (Table 3), the main factors associated with RSP-Pnc carriage were daycare center attendance (adjusted OR [aOR] = 1.55, 95 % CI [1.36;1.77]), age < 1 year old (aOR = 1.19, 95 % CI [1.04;1.35]), and antibiotic use within 3 months (aOR = 1.78, 95 % CI [1.56;2.03]. In contrast, decreased RSP-Pnc carriage was associated with presence of siblings and post-PCV13 period (aOR = 0.75, 95 % CI [0.65;0.85]) and (aOR = 0.71, 95 % CI [0.62;0.81]). Factors associated with Hi carriage were conjunctivitis (aOR = 4.10, 95 % CI [3.54;4.76]), bilateral AOM (aOR = 1.33, 95 % CI [1.17;1.51]), daycare center attendance (aOR = 1.93, 95 % CI [1.70;2.19]), age < 1 year old (aOR = 1.49, 95 % CI [1.31;1.69]), presence of siblings aOR = 1.82, 95 % CI [1.60;2.07]), and post-PCV13 period (aOR = 1.23, 95 % CI [1.05;1.44]). Post-PCV13 period was the only factor retained in the ß-lactamase–producing Hi carriage model and was associated with decreased carriage (aOR = 0.65, 95 % CI [0.54;0.79]).

**Table 3:** Risk factors of nasopharyngeal carriage of Pnc, strains with reduced susceptibility to penicillin (RSP) Pnc, Hi and ß-lactamase + Hi strains by univariate and multivariate analysis

	Univariate analysis			Multivariate analysis		
	OR	95 % CI	*P* value	aOR	95 % CI	*P* value
Carriage of Pnc*						
Otalgia + fever ≥38.5 °C	1.39	1.26;1.53	<10^−4^	1.29	1.17;1.42	<10^−4^
Daycare centre	1.33	1.21;1.46	<10^−4^	1.61	1.46;1.78	<10^−4^
Siblings	1.32	1.20;1.45	<10^−4^	1.45	1.31;1.60	<10^−4^
Age <1 year	1.03	0.94 ;1.14	0.48	1.04	0.95 ;1.15	0.39
Recent use of cephalosporin	0.98	0.86;1.11	0.74			
Bilateral AOM^a^	0.94	0.84;1.06	0.33			
Otitis-prone children^b^	0.91	0.79;1.04	0.16			
Periods (post-PCV13)	0.86	0.78;0.94	0.002	0.84	0.77 ;0.93	0.01
History of AOM^b^	0.86	0.77;0.96	0.005			
Hi carriage	0.79	0.72 ;0.86	<10^−4^			
Recent use of antibiotics	0.78	0.71 ;0.86	<10^−4^	0.73	0.66 ;0.81	<10^−4^
Conjunctivitis	0.47	0.42;0.52	<10^−4^	0.47	0.42;0.52	<10^−4^
Carriage of RSP Pnc**						
Recent use of antibiotics	1.88	1.66 ;2.14	<10^−4^	1.78	1.56 ;2.03	<10^−4^
Day care centre	1.68	1.48;1.91	<10^−4^	1.55	1.36 ;1.77	<10^−4^
Recent use of cephalosporin	1.63	1.38;1.92	<10^−4^			
Otitis-prone children^b^	1.60	1.33;1.93	<10^−4^			
History of AOM^b^	1.58	1.36;1.83	<10^−4^			
Bilateral AOM^a^	1.15	0.98;1.35	0.08			
Age <1 year	1.08	0.96 ;1.23	0.21	1.19	1.04 ;1.35	0.009
Conjunctivitis	1.08	0.93;1.26	0.33			
Hi carriage	0.95	0.84 ;1.08	0.46			
Otalgia + fever ≥38.5 °C	0.93	0.82;1.06	0.27			
Periods (post-PCV13)	0.72	0.63 ;0.81	<10^−4^	0.71	0.62 ;0.81	<10^−4^
Siblings	0.70	0.62 ;0.80	<10^−4^	0.75	0.65 ;0.85	<10^−4^
Carriage of Hi***						
Conjunctivitis	4.22	3.76 ;4.74	<10^−4^	4.10	3.54 ;4.76	<10^−4^
Daycare centre	1.74	1.58 ;1.91	<10^−4^	1.93	1.70;2.19	<10^−4^
History of AOM^b^	1.65	1.48 ;1.83	<10^−4^			
Otitis-prone children^b^	1.54	1.34 ;1.77	<10^−4^			
Siblings	1.49	1.36 ;1.64	<10^−4^	1.82	1.60;2.07	<10^−4^
Bilateral AOM^a^	1.47	1.31 ;1.65	<10^−4^	1.33	1.17 ;1.51	<10^−4^
Recent use of antibiotics	1.42	1.29;1.56	<10^−4^	1.28	1.12;1.45	<10^−4^
Age <1 year	1.37	1.25 ;1.50	<10^−4^	1.49	1.31;1.69	<10^−4^
Recent use of cephalosporin	1.19	1.05 ;1.35	0.005			
Periods (post-PCV13)	1.18	1.08;1.29	<10^−3^	1.23	1.05;1.44	0.01
Otalgia + fever ≥38.5 °C	0.84	0.76;0.92	<10^−3^			
Pnc carriage	0.79	0.72 ;0.86	<10^−4^			
Carriage of ß-lactamase producing Hi****						
Otitis-prone children^b^	1.27	0.99 ;1.64	0.06			
Recent use of cephalosporin	1.21	0.96 ;1.53	0.11			
History of AOM^b^	1.18	0.94;1.48	0.16			
Recent use of antibiotics	1.17	0.97 ;1.40	0.11			
Age <1 year	1.16	0.96;1.40	0.12	0.86	0.72;1.04	0.13
Conjunctivitis	1.10	0.91 ;1.33	0.32			
Bilateral AOM^a^	0.96	0.76 ;1.23	0.76			
Otalgia + fever ≥38.5 °C	0.94	0.78;1.14	0.52			
Siblings	0.91	0.75 ;1.10	0.33			
Daycare centre	0.90	0.75;1.08	0.27			
Pnc carriage	0.89	0.74 ;1.08	0.24			
Periods (post-PCV13)	0.65	0.54 ;0.78	<10^−4^	0.65	0.54 ;0.79	<10^−4^

aOR, adjusted odds ratio; 95 % CI, 95 % confidence interval; Pnc: *pneumococcus*; Hi*: H. influenzae*; AOM: Acute otitis medi; a ^a^data not available in 2006/2008, ^b^data not available in 2006/2007

*univariate and multivariate analysis of overall population (*n* = 7,200), with 4,033 Pnc carriers; **univariate and multivariate analysis of Pnc carriers (*n* = 4,033), with 1,735 RSP Pnc carriers; ***univariate and multivariate analysis of overall population (*n* = 7,200), with 3,624 Hi carriers; ****univariate analysis of Hi carriers (*n* = 3,624), with 519 ß-lactamase+

## Discussion

The levels of resistance to antibiotic for *S. pneumoniae* and *H. influenzae* are the cornerstones of the rationale for antimicrobial recommendations for AOM [5, 8]. In 2011, guidelines in France designated amoxicillin as the first-line drug for AOM requiring antibiotics, with amoxicillin-clavulanate and cefpodoxime proxetil limited to rare and specific situations [3]. These guidelines were based on a decreased proportion of ß-lactamase–producing Hi strains in France [13]. Complying with these 2011 recommendations, in 2012, the most frequently (66 %) prescribed antibiotic for AOM in France was amoxicillin, as was recently shown [14, 15]. Conversely, prescriptions of broad-spectrum antibiotics such as amoxicillin-clavulanate and cefpodoxime proxetil sharply decreased [14, 15].

Since 2011 (Fig. 1), the NP carriage of RSP-Pnc decreased 18 % in the post-PCV13 period. Moreover, this reduction was associated with a decrease (−20 %) in the carriage of ß-lactamase–producing Hi strains. Accordingly, amoxicillin as the first-line drug for AOM requiring antibiotics remains an adapted recommendation in France. In addition, we did not identify any risk factor associated with carriage of ß-lactamase–producing Hi strains (such as daycare center attendance, otitis-prone condition or recent antibiotic use). Taking into account the low proportion of ß-lactamase–producing Hi strains and the lack of risk factors associated with their carriage, the prescription of amoxicillin-clavulanate as the first-line drug for AOM in this situation seems to have no benefit or justification.

The decrease in antibiotic resistance to *S. pneumoniae* was expected [21]. As in other studies, the reduction is linked to the 66 % decrease in the 6 additional PCV13 serotypes in our study [21, 22]. Despite a dramatically decrease of vaccine serotypes, 19A known in France to harbor a high proportion of RSP remained frequently isolated in post PCV13 period (8.4 %) [23]. Several non-vaccine serotypes such as 15B/C (12.2 %), 15A (9.3 %), 11A (9.1 %), 35B (7.5 %), 23A (5.9 %), and 6C (5.0 %) seem to emerge in the post PCV13 period.

In contrast, the decrease in ß-lactamase–producing Hi strains was not expected. Two explanations could be raised. The first is the reduced antibiotic use for children in France. Since 2001, following a national campaign promoting a judicious use of antibiotics in France, antibiotic use in children has been sharply reduced, particularly for children < 2 years [24]. Furthermore, PCV implementation may have led to an additional reduction in prescriptions [25]. The second hypothesis is more speculative: in our population, most Hi strains now produce biofilms [26], which allows for resistance to antibiotic treatments without another mechanism of resistance required [27, 28].

PCV13 impact in our population was expected since we have already showed the impact of PCV7 on carriage and antibiotic resistance [11]. We have previously showed in pre PCV13 period that Pnc carriage was less frequently associated with AOM treatment failure than Hi [29]. However, in this current study, we have not analyzed the evolution of risk of AOM antibiotic failure between pre and post PCV13 periods.

This situation of infection and antibiotic resistance is very dynamic and the few non-vaccine strains of *S. pneumoniae*, which are resistant to penicillin, may, similar to serotype 19A, become more prevalent. This possibility underscores the importance of the continuous availability of current data that reflects local and national microbiologic trends.

## Conclusion

The NP carriage rate of RSP-Pnc strains and ß-lactamase–producing Hi strains has decreased in children with AOM in France since 2010, the year of PCV13 implementation in France. Accordingly, amoxicillin as the first-line drug for AOM requiring antibiotics remains a valid recommendation. However, the AOM microbiology is evolving and requires continuous monitoring and adjustments of policy for antibiotics.
